# Florid cemento-osseous dysplasia: Report of 2 cases

**DOI:** 10.4317/jced.55288

**Published:** 2018-11-01

**Authors:** Jorge Toledano-Serrabona, Sergio Núñez-Urrutia, Erika Vegas-Bustamante, Alba Sánchez-Torres, Cosme Gay-Escoda

**Affiliations:** 1 Student in Dental Degree. School of Medicine and Health Sciences, University of Barcelona, Barcelona (Spain) 2 DDS, MS. Master’s Degree Program in Oral Surgery and Implantology, School of Medicine and Health Sciences, University of Barcelona (Spain); 2 DDS, MS. Master’s Degree Program in Oral Surgery and Implantology, School of Medicine and Health Sciences, University of Barcelona (Spain); 3 DDS, MS. Associated professor in Oral Surgery. Master’s Degree Program in Oral Surgery and Implantology, School of Medicine and Health Sciences, University of Barcelona. Researcher of the IDIBELL institute, Barcelona (Spain); 4 DDS, MS. Associated professor in Oral Surgery. Master’s Degree Program in Oral Surgery and Implantology, School of Medicine and Health Sciences, University of Barcelona. Researcher of the IDIBELL institute, Barcelona (Spain); 5 MD, DDS, MS, PhD, EBOS, OMFS. Chairman and Professor of Oral and Maxillofacial Surgery, School of Medicine and Health Sciences, University of Barcelona. Director of the Master’s Degree Program in Oral Surgery and Implantology (EFHRE International University/FUCSO). Coordinator/Researcher of the IDIBELL Institute. Head of the Oral Surgery, Implantology and Maxillofacial Surgery Department of the Teknon Medical Center, Barcelona (Spain)

## Abstract

**Introduction:**

Florid cemento-osseous dysplasia is a non-neoplastic fibro-osseous lesion which often has an asymptomatic slow growth. Unfortunately, these lesions are usually diagnosed through routine radiographic examination. The aim of this study was to describe the main clinical, radiological and histological characteristics of two case reports diagnosed with florid cemento-osseous dysplasia.

**Case reports:**

Two cases of florid cemento-osseous dysplasia with different clinical and radiological features were presented. Panoramic radiographs showed multiple radiopacities compatible with fibro-osseous lesions in distinct areas of the maxillary bones. The histological study revealed a sclerotic mass which continued imperceptibly with root cement with scarce fibrous lax tissue.

**Conclusions:**

The replacement of healthy bone by metaplastic bone and fibrous tissue is the main histological feature. Therapeutic abstention with active clinical and radiographic control visits is recommended in asymptomatic cases.

** Key words:**Fibro-osseous lesions, cemento-osseous dysplasia, florid cemento-osseous dysplasia, gigantiform cementoma, osseous dysplasia.

## Introduction

Fibro-osseous lesions of the head and neck were first described by Lichtenstein in 1938 (1). Since then, various attempts have been carried out to classify this wide range of lesions by different researchers and scientific societies although it is still a controversial issue (1-6).

It has been historically difficult to find agreed diagnostic criteria to define these lesions. The first World Health Organization (WHO) classification made in 1971 (3) included the term “gigantiform cementoma” in the group of fibro-osseous lesions. Melrose *et al.* (4) in 1976 used the term of cemento-osseous dysplasia (COD) to define a set of dense, radio-opaque and lobulated masses, similar to cementum scattered over different maxillary zones.

The second classification made by the WHO in 2005 (5) suggested the term “florid cemento-osseous dysplasia” (FCOD) to replace the old term and defined the periapical cemento-osseous dysplasia (PCOD) as an independent lesion.

Finally, the current classification of head and neck tumors published by the WHO in 2017 sorted the CODs lesions into three groups: periapical COD, focal COD and florid COD (6).

CODs are non-neoplastic fibro-osseous lesions that have a long and asymptomatic course and whose diagnosis results from a radiologic finding (2,6,7). Histologically, a replacement of healthy bone by metaplastic bone and fibrous tissue is observed (7,8).

The aim of this study was to describe the main clinical, radiological and histological characteristics of two case reports diagnosed as florid cemento-osseous dysplasia.

## Case Reports

-Case 1

A 42-year-old Moroccan female patient, with no relevant medical background, was referred to the Oral Surgery Department of the University of Barcelona to assess a lesion compatible with a tumor associated with tooth 4.7. The patient reported an acute and pulsating 1-month-long pain on the fourth quadrant that became more severe after a previous attempt of extracting tooth 4.7, one week before being referred to us. Facial exploration confirmed an ipsilateral tumefaction. Both mouth opening and lateral mandibular movements were found to be diminished. Cervical palpation revealed a one-centimeter-in-diameter right submandibular adenopathy, with a soft consistency, which was not adhered to deep regions and which caused discomfort to palpation. Intra-oral examination revealed a decay in tooth 4.7.

A pharmacological oral treatment was prescribed based on 1 tablet of Augmentine plus ®, 1000 mg/ clavulanic acid, 62. 5 mg (Glaxo Smithklyne; Madrid; Spain) every 8 hours for seven days, 1 capsule of magnesic metamizol, 575 mg (Khern Pharma EFG®; Madrid; Spain) every eight hours for seven days, 1 capsule of ibuprofen 600 mg (Normon®; Madrid; Spain) every 8 hours for 7 days and one daily capsule of omeprazol 40 mg (Sandoz®; Barcelona; Spain) in the mornings.

**Figure 1:**
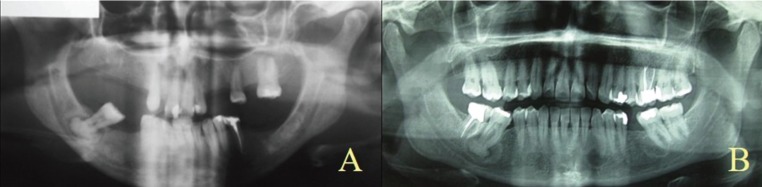
Orthopantomography. A) Case 1. Radio-opaque lesions in mandible localized on the periapex of tooth 4.7, the edentulous area corresponding to tooth 3.6 and 3.7 and periapical radiolucent lesion periapical of tooth 1.1. B) Case 2. Periapical radio-opaque lesion of tooth 4.7 and periapical radiolucent lesions in teeth 3.3 and 3.7.

An orthopantomography (OPG) and a computerized tomography (CT), which included a 3-dimensional reconstruction of both jaws, were ordered. Radiological imaging revealed a radio-opaque lesion of 1.5 cm surrounded by a sclerotic halo that comprised the apices of tooth 4.7. Furthermore, numerous sclerotic lesions were observed in edentulous sections in those positions concerning teeth 4.6, 3.6, and 3.7, as well as a periapical radiolucent lesion in tooth 1.1 (Fig. 1A). Vitality test for teeth resulted positive, except for 4.7, which showed signs of pulp necrosis. Periodontal examination of 4.7 confirmed the presence of probing depths of more than 6 mm in three vestibular probing sites but without dental mobility. The rest of teeth had physiological probing depths.

The suggested treatment included the surgical extraction of tooth 4.7 and the elimination plus curettage of the residual bone cavity and histological exam of the lesion.

**Figure 2:**
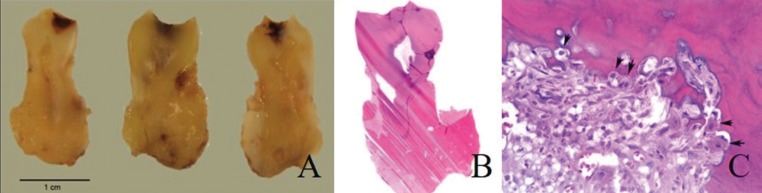
Macroscopic and microscopic appearance of Case 1. A) Serialized sections of a tumor formation in relation to tooth roots. B) Histological cut revealing a sclerotic bone mass in relation to the radicular cementum of the molar tooth, in the absence of periodontal ligament. Hematoxylin-Eosin (HE). C) Detailed fragment around the lesion, with osteoblastic rim (see arrows). The adjacent fibrous tissue presents an infiltrate of polymorphonuclear neutrophils (HE, 400x).

Serial cuts on the tooth revealed a significantly thickened root (Fig. 2A). Histological tests reported a sclerotic mass that extended to the radicular cementum imperceptibly. There was a scarce amount of lax fibrous tissue within the bone (Fig. 2B), while some areas showed an intense inflammatory infiltration made up of polymorphonuclear neutrophils. Osteoblastic cells were focally observed in the surrounding tissue, encircling some of the bone trabeculae (Fig. 2C). The anatomopathologic study established the final diagnosis of COD. Clinical and radiological control at 6 months evidenced a correct scarring process of both soft and bone tissues.

-Case 2

A 37-year-old female patient from Morocco with no relevant medical background presented to the Department of Oral Surgery for the assessment and management of numerous mandible fibro-osseous lesions. The patient did not mention previous pain episodes. Vitality tests on the teeth involved were positive, except for tooth 3.7.

The OPG revealed a radio-opaque 1.5-cm-in-diameter lesion surrounding the roots of tooth 3.7, whose perimeter was delimited by a radiolucent halo. Numerous lesions with a similar size were observed around the apices of teeth 3.3, 3.5 and 3.6 (Fig. 1B). A CT was conducted to assess size and confirm the extension of lesions. Endodontic treatment and periapical surgery plus enucleation of the lesion were conducted on tooth 3.7.

**Figure 3:**
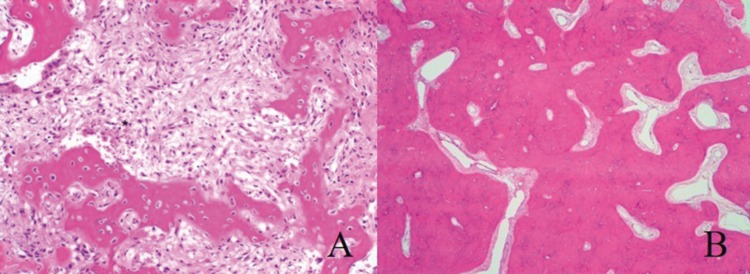
Histological study of Case 2. A) Disorganized bone with fibrous tissue background and hemorrhagic points (*) (HE, 200x). B) Sclerotic bone area with almost no fibrous tissue (HE, 40x).

The histological study showed hypercementosis and gutta-percha filling the pulp cavity. The tissue was found to have areas made up of laminar or disorganized bone, and inter-trabecular stroma made up of fibrous tissue with spindle-shaped fibroblasts and absence of cytological atypia. Focally, the lesion presented abundant capillaries and blood extravasation areas (Fig. 3A). The presence of sclerotic bone with scarce fibrous tissue could be noticed in some areas (Fig. 3B). The histological result was FCOD. Suture was removed at 7 days but a purulent exudate into the wound required the administration of Augmentine® (amoxicillin 875 mg/ clavulamic acid, 125 mg; Glaxo Smithklyne; Madrid; Spain), 1 tablet every 8 hours for 7 days. A control visit carried out at day 15 showed an adequate soft tissue restoration and complete absence of infection.

## Discussion

FCOD is an infrequent COD that presents numerous radio-opaque lesions which can affect 2 or more maxillary quadrants. FCODs are generally expansive, presented with pain and discharge secondary to infection (2,4,6,7,9,10). Sedano *et al.* (11), reported FCOD variants that could be inherited in the form of autosomal dominant pattern and variable phenotypic expression, although the cases described here did not include familiar FCOD history.

According to the last WHO classifications (5,6), some histological singularities point the periodontal ligament to be a possible etiologic factor, due to the continuity between the laminar bone and the radicular cementum (9). In Case 2, no close link could be established between the tooth and the lesion produced after the elimination. Yet, the histological study observed hypercementosis on the radicular surface of the tooth as well as the formation of laminar bone in contact with the roots.

FCOD has a clear predominance for female patients. Around 90% of cases are diagnosed between the third and fourth decade of life (5,6). Black women seem to be the most affected group, which coincides with our cases, followed by Asian women, as well as Caucasians in a lower proportion (2,7,10,12).

Therapeutic abstention and periodical controls are preferred in asymptomatic FCODs (2,9,10,12). A strict control is recommended in patients using a removable prosthesis. Otherwise, a poorly adapted prosthesis could lead to the resorption of alveolar bone and contribute to the exposition of sclerotic masses to the oral medium (9,10,12). Moreover, FCOD patients with periodontal involvement should undergo periodical controls. Both groups of patients have a high probability of suffering infectious complications (8,13).

However, when lesions lead to deformations or functional alterations, it is recommended to perform some bone remodeling rather than using wide resection techniques due to the risk of fracture or post-operative infections (2,7).

FCOD tends to have a scarce vascularization, which paves the way for the infection of these lesions. Any uncontrolled FCOD infection may lead to a chronic diffuse osteomyelitis (7). Some authors do not recommend surgical treatment in asymptomatic patients (10,13) unless symptoms appear (14). Periapical surgery achieves good results in the treatment of fibro-osseous lesions (10,14). Some literature precedents are in favor of mixed treatments (endodontic and surgical procedures) for the management of FCODs. Case 2 received periapical surgery, which made possible to study the sample and preserve the tooth. An infection process that was detected in a post-operative control was eventually solved.

Images concerning both cases (Fig. 1A,B) show different radiographic aspects that vary from radiolucent to radio-opaque images as this condition suffers progressing stages. In the first stage, both fibrous tissue and immature medullary bone replaced the healthy bone to become a compact laminar bone similar to cementum (radio-opaque cortical bone) (7,13,15).

The occurrence of numerous fibro-osseous lesions should include the following nosologic disorders into differential diagnosis: odontogenic cysts, cementoblastoma, osteoma, odontogenic keratocystic, fibrous dysplasia, ossifying fibroma, Paget’s disease, Gardner syndrome, osseous metastases, among others (2).

Sometimes, the biological unspecificity of CODs due to its progressive stages, makes difficult to achieve a diagnostic of CODs. Definitive diagnosis is the result of a histological study supplemented with previous clinical and radiological characteristics.
